# Endometrial immune assessment in patients with a history of previous euploid blastocyst failure

**DOI:** 10.3389/fimmu.2025.1547159

**Published:** 2025-04-07

**Authors:** Jemma Garratt, Baharak Mohammadi, Balsam Al-Hashimi, Elena Linara-Demakakou, Rukma Bhattacharya, Kamal K. Ahuja, Nick Macklon, Mona Rahmati

**Affiliations:** ^1^ Department of Reproductive Medicine, London Women’s Clinic, London, United Kingdom; ^2^ School of Biosciences, University of Kent, Canterbury, United Kingdom

**Keywords:** endometrial immune profiling, embryo implantation, embryo implantation failure, euploid embryo, PGT-A

## Abstract

**Background:**

Influx and establishment of key endometrial immune factors in the mid-luteal phase of the menstrual cycle is paramount for successful embryo implantation. Endometrial immune dysregulation is associated with repeated embryo implantation failure and miscarriage. In *in vitro* fertilisation cycles, approximately 30% of embryos diagnosed as chromosomally normal will still fail to produce a viable live birth, yet factors such as the endometrium are rarely clinically explored.

**Methods:**

In this retrospective analysis, clinical outcomes were compared between patients undergoing their first euploid transfer in a conventional substituted cycle (n=612), patients undergoing a euploid transfer in a similar cycle after previous euploid failure (n=149) and the study group of patients with previous euploid transfer failure who received a modified endometrial preparatory regimen following endometrial immune profiling targeting uterine natural killer cell recruitment, maturity and activity as well as their key regulatory counterparts (n=37).

**Results:**

Significant differences were found between first euploid attempt outcomes and patients with previous failures who didn’t use endometrial testing (implantation rate 63% vs 51, *P*=0.02; clinical pregnancy rates 55% vs 40%, *P*=0.002; live birth rates 50% vs 38%, *P*=0.02). Patients with previous failures who underwent endometrial immune profiling and a subsequent personalised plan exhibited a trend towards improved clinical outcomes than those with previous failures and no testing (implantation rate 65% vs 51%; clinical pregnancy rate 57% vs 40%; live birth rate 54% vs 38%, respectively) although statistical significance was not demonstrated. Clinical outcomes were comparable between the endometrial immune profiling group and those undergoing a first euploid attempt (implantation rate 65% vs 63%; clinical pregnancy rate 57% vs 55%; live birth rate 54% vs 50%, respectively).

**Conclusions:**

Patients who had a failed attempt when using a euploid embryo had lower chances of pregnancy when repeating their treatment, unless they received a personalised endometrial preparation regimen derived from the results of endometrial immune profiling. These preliminary findings indicate the potential value of guiding management based on immune endometrial testing.

## Introduction

The central dogma for establishing a pregnancy is the arrival of a viable competent embryo into the uterus when the endometrium is receptive. This biological event is essential for implantation to commence; aberrances in either embryo or endometrium may ultimately lead to implantation failure and embryo rejection.

Treatment with *in vitro* fertilisation (IVF) aims to overcome reproductive challenges for patients. A major factor affecting patients’ ability to conceive from IVF is oocyte-derived aneuploidies which increase in incidence with age, reaching 66% by 40-42 years (1). A clinical technique used to ameliorate the clinical impact of this is Pre-Implantation Genetic Testing for Aneuploidy (PGT-A), which aims to determine chromosomal copy number from biopsied trophectodermal cells of a day 5 blastocyst. Recent non-selection studies have shown that embryos known to be chromosomally abnormal, referred to as aneuploid, rarely result in viable births. In a review of five non-selection studies, embryos diagnosed as aneuploid were reported to have a 98% positive predictive value of embryo lethality (2). Most non-viable embryos will usually fail to implant, some may succeed but result in miscarriage (3, 4). Evidence that encompasses both the embryo and the endometrium’s role in implantation suggest that an aneuploid or poorly viable embryo can secrete distinct molecules that are detected by the endometrial stroma, preventing further maternal investment in a non-viable pregnancy. (5, 6). This suggests there may be a dual function in modulating acceptance of the embryo into the endometrium.

Despite the use of techniques to eliminate aneuploidies, patients still fail to conceive. With 30% of euploid transfers in IVF failing to implant, other factors such as the endometrium could be attributable (7). However clinically, the complexity of the endometrium and its function in mediating embryo implantation and sustained pregnancy is poorly recognised. Routine endometrial investigations rarely surpass ultrasound scans and measuring endometrial thickness, with some routine measurements of serum progesterone levels during luteal phase support. Proper investigation of the endometrium is thus relatively disregarded and any treatments therefore tend to be empirical.

The concept of endometrial receptivity is based on the distinct transformation of the endometrium in its secretory phase. Aside from development into a glandular structure, an influx of immune cells and immunomodulatory factors establish a balance of both pro-inflammatory and pro-modulatory effects. These milieus play a key role in ensuring the endometrium is in a state that is receptive to an implanting blastocyst. Within the very complex local uterine immune milieu, involving multiple cell types (uNK, regulatory T-cells, dendritic cells) and cytokines (Th1, Th2, Th17), the Th1/Th2 balance has a key role. Pro-immunomodulatory Th2 cells are considered to provide protection to the foetus, while regulating pro-inflammatory Th1 cytokines (8, 9). While Th1 cells and their cytokines in excess are thought to be embryonically cytotoxic, a tightly-controlled concentration is required to promote trophoblastic invasion into the endometrium during implantation (10, 11).

Unique in their distinctive CD56^bright^, CD16^dim^ antigen expression, uterine Natural Killer (uNK) cells exert key functions at the time of implantation in the endometrium and are crucial for governing endometrial receptivity in the mid-luteal immune milieu (12–14). Unlike circulating peripheral NK cells, uNKs are within an immune niche regulated by elevating mid-luteal progesterone concentrations, and by specific local cytokines within the endometrium (15). Namely, local secretions of IL-15 recruit uNK cell populations, while IL-18 promotes uNK cell maturation and activity. Thus uNK cells are required to establish a balanced pseudo-inflammatory environment in the decidua (16).

Early hypotheses suggested that changes in uNK cell counts, recruitment and maturity may be potential causes for implantation failure or miscarriage. Indeed, in patients experiencing recurrent embryo implantation failures (RIF) (17, 18) and recurrent miscarriages (RM) (19), significant differences in the proportion of uNK cells and their associated markers have been noted (18, 20). uNK cell immunomodulation depends on two key cytokines, IL-18 and IL-15 (21). More recently, modulators of these cytokines were unveiled, namely transmembrane protein TWEAK and its respective ligand Fn-14. These act to immunomodulate uNKs by inhibiting Th1-driven differentiation of uNK cells into cytotoxic cells and neutralise high IL-18 concentrations (22).

In light of these findings, development of a clinical diagnostic test that profiles the mid-luteal phase endometrial immune milieu, specifically utilising uNK cell count, maturation and key associated immunomodulatory molecules (17, 23), found imbalances in these markers in 80% of RIF and RM patients (19, 24). Moreover, subsequent treatment cycles aiming to correct the dysregulation reported promising results, with increased LBRs (19, 24).

Within this study, we aim to explore whether patients who had at least one embryo implantation failure or miscarriage with a euploid blastocyst clinically benefit from endometrial immune profiling and a subsequent personalised treatment cycle, in terms of achieving both embryo implantation and a live birth, in comparison to those that did not undergo endometrial immune profiling prior to a euploid blastocyst transfer and had a conventional treatment cycle.

## Materials and methods

### Patients/participants

This was a retrospective cohort study of 609 patients who underwent PGT between 2019-2024 at a single UK-based centre. Inclusion criteria were patients who created blastocysts through IVF or ICSI and underwent PGT between January 2019- January 2024 via Next Generation Sequencing (NGS) and generated at least one euploid blastocyst. Further inclusion criteria were those who transferred a vitrified-warmed euploid blastocyst that resulted in implantation failure or miscarriage and subsequently underwent further euploid transfers, with or without endometrial immune profiling. Exclusion criteria were patients who underwent immune profiling but did not follow the treatment suggested or transferred outside of the test validity, array CGH-tested PGT embryos and non-euploid embryos (mosaics or no result). Any patients with pre-existing autoimmune diseases or taking immunosuppressants or steroids were further excluded.

### Embryo biopsy & chromosomal analysis via PGT-A

On day 5, 6 or 7 of embryo culture, 5-10 cells of the blastocyst trophectoderm were biopsied using the laser technique, placed in sterile tubes and sent for NGS (25), as described previously (CooperGenomics, UK). Only patients that transferred blastocysts diagnosed as euploid were included in this study. Mosaic and no result embryos were excluded from analysis.

All blastocysts were morphologically assessed using a validated modified Gardner system for up to day 3 embryos, and the ACE/NEQAS grading system for day 5 blastocysts (26, 27). All embryos were cultured in EmbryoScopes (EmbryoScope time-lapse system - Vitrolife) (Vitrolife, UK) (28). Euploid blastocysts were prioritised for transfer, but where patients had more than one euploid blastocyst, these were secondarily prioritised for transfer by morphology and time-lapse morphokinetics (27).

### Endometrial assessment

Criteria for referral for endometrial immune profiling was as follows. Patients who had at least one previous failed euploid transfer were eligible for referral for testing. Exclusion criteria were patients with poor morphological blastocyst quality or history of a thin endometrial lining.

All endometrial biopsies were performed in a mock substituted cycle, with oestradiol 8-10mg daily (Progynova, Bayer plc, UK). Luteal support was given as micronised vaginal progesterone 400mg three times daily (Cyclogest, L.D. Collins & Co Ltd., UK). Endometrial biopsy was performed with or without sedation following five days of progesterone with a pipelle. Endometrial tissue was stored in RNA later for transport.

All biopsies were sent for immunological profiling, as described previously (Matrice Lab Innove, Paris, France) (18, 20). To summarise, immune profiling analysed variables such as uNK cell count, recruitment and activation by testing for key markers such as IL-18/TWEAK mRNA, IL-15/Fn-14 and CD56^+^ counts using RT-qPCR.

Patients could receive one of four results:

1. “Balanced”- characterised by IL-18/TWEAK and IL-15/Fn-14 mRNA ratios and CD56^+^counts within the same range as defined by a fertile cohort.
2. “Overactive”- characterised by high IL-18/TWEAK and/or IL-15/Fn-14 mRNA ratios and/or a high CD56^+^count.
3. “Underactive”- characterised by IL-15/Fn-14 mRNA ratios and/or a low CD56^+^count and/or a very low local IL-18/TWEAK mRNA ratio.
4. “Mixed”- characterised by a high ratio of IL-18/TWEAK mRNA in tandem with low IL-15/Fn-14 mRNA ratio (20).

### Endometrial preparation for frozen embryo transfer

All patients referred for endometrial assessment were prescribed a substituted frozen embryo transfer (FET) cycle based on their immune profiling results, as described previously (20). The protocols for each immune profile are as follows:

1. Balanced: Underwent a standard substituted cycle, commencing oestradiol on cycle day 2-3 until endometrial thickness measured 8mm, followed by 5 days of progesterone pessary administration 400mg 3x daily.
2. Overactive: Corticoids were prescribed at the dose of 20mg daily, with the aim to decrease Th1 cytokines and uNK cytotoxicity, reduce IL-15 overexpression and modulate Th1/Th2 cytokine ratios. Oral oestradiol was prescribed to downregulate IL-18 in cases displaying overexpression. High doses of progesterone were administered by dual route (vaginal and intramuscular/subcutaneous) to exert an immunosuppressive function.
3. Underactive: An endometrial scratch was performed during the mid-luteal phase of the preceding cycle. Supplementation with hCG was recommended on day 4, 6 and 8 after starting progesterone in cases with low CD56^+^ mobilisation or uNK cell immaturity.
4. Mixed: An endometrial scratch was performed and hCG was administered on days 4, 6 and 8 following commencement of progesterone. Patients were also prescribed corticoids at a dose of 10mg daily and oral oestradiol (20).

Only single euploid embryo transfers were performed following immune profiling and personalised plan.

Patients who did not undergo endometrial assessment, comprising the control group, underwent a standard substituted FET cycle, commencing oestradiol 8mg daily (Progynova, Bayer plc, UK) on cycle day 2-3 until endometrial thickness measured 8mm or above via transvaginal ultrasonography, with all euploid blastocysts transferred following 5 days of progesterone administration (Cyclogest, L.D. Collins & Co Ltd., UK).

### Outcome definitions

Primary outcome measure was implantation rate (IR), defined as a positive serum βhCG test. Evolution of pregnancy was confirmed with viable intrauterine scans/foetal hearts at 7 and 12-weeks gestation. Secondary outcome measures were clinical pregnancy rate, defined as confirmation of foetal heart at 7 weeks via ultrasound scans, and live birth rate (LBR). Another outcome calculated was miscarriage rate (MR), defined as the total number of pregnancy losses between a positive βhCG test and 12 weeks gestation. Outcome measures are calculated as per embryo transferred.

### Statistical analyses

Univariate analyses comprised of non-parametric and parametric t-tests for continuous variable comparisons, with data reported as median [interquartile range (IQR)] for non-normally distributed variables and mean ± SD for normal distributions. Distribution normality was tested via histograms and Shapiro-Wilk tests. For comparison of multiple non-normally distributed continuous variables, Kruskal-Wallis tests were used for univariate analyses. For multiple normally distributed continuous variables, ANOVAs were used. For categorical variables, Fisher’s Exact tests were applied to compare proportional rates in small sample sizes. *Post-hoc* comparison tests were conducted for results returning with significance. *P*<0.05 denoted statistical significance. All analyses were conducted on R. 4.3.1.

### Ethical approval

Since this was a retrospective service evaluation using anonymised clinical data further ethical approval by an IRB was not considered to be required by the University of Kent Central Research Ethics Advisory Group.

## Results

A total of 609 patients underwent 640 PGT cycles in which at least one euploid blastocyst was transferred between 2019-2024. This first euploid transfer (n=609) comprises the first comparative group for this study (First attempt group). Following a first euploid blastocyst transfer attempt, 301 patients’ outcome resulted in implantation failure or miscarriage (49%). All patients who had a miscarriage were tested for the usual causes of miscarriage (thrombophilia, dysthyroidism, diabetes, uterine cavity assessment). Of these, 173 patients (57%) underwent at least one subsequent euploid embryo transfer after failure. Following an adopted clinical policy, patients were offered to undergo immune profiling of the endometrium after undergoing at least one failed euploid transfer. Overall, 50 patients were referred for immune profiling after one or more euploid transfer failures, of which 37 completed the treatment (Previous euploid failure/s [PEF] + endometrial immune profiling [EIP] group). In total, 149 patients underwent a second euploid transfer without undergoing immune profiling, comprising a second comparison group (PEF + no EIP group). Some patients underwent multiple euploid embryo transfer failures prior to being referred for immune profiling. There is therefore patient cross-over present between groups, where some patients underwent a second euploid transfer attempt without immune profiling, and then underwent immune profiling for a subsequent attempt.

The mean oocyte age in this cohort (n=640) was 36.7 ± 4.2 with a mean patient age of 37.8 ± 4.1. Overall, 95% (608/640) of cycles used own oocytes and 5% (32/640) with donor oocytes. The cohort had a median AMH of 17.9 (1.1, 34.8) and median BMI of 24 (18.5, 30). Characteristics stratified by group can be found in **Table 1**. Overall, there was a statistically significant difference in the oocyte age across all groups (*P*<0.001), with *post-hoc* analyses revealing significant differences between both the first attempt and PEF + no EIP group (36.8 ± 4.2 vs 35.8 ± 4.1, *P*=0.03) and between first attempt and the PEF + EIP group (36.8 ± 4.2 vs 37.8 ± 4.1, *P*=0.03). All other characteristics did not differ between groups (**Table 1**).

**Table 1:** Patient and PGT cycle characteristics between 2019-2024.

Patient/cycle characteristics	First attempt, euploid transfer (n=609)	Previous euploid failure + no immune profiling (n=149)	Previous euploid failure + immune profiling (n=37)	P-value
Oocyte age, mean ± SD	36.8 ± 4.2	35.8 ± 4.1	37.8 ± 4.1	*P*<0.001
Patient age, mean ± SD	37.8 ± 4.1	37.5 ± 4.5	38.6 ± 3.7	*P*=0.3
BMI (kg/m^2^), median [IQR]	24 [19, 30]	26.5 [19, 31]	22.8 ± [19, 28]	*P*=1
AMH (pmol/L), median [IQR]	17.4 [0.6, 34.2]	18.1 [3.4, 32.8]	17.9 [3.8, 32]	*P*=1
Own oocytes, n (%)	95% (583/612)	91% (138/149)	90% (35/37)	*P*=0.4
Donor oocytes, n (%)	5% (29/612)	9% (11/149)	10% (2/37)	–

-Statistical test not applicable.

The median number of oocytes collected per fresh IVF collection cycle was 13 [IQR 3, 23]. In total, 9620 oocytes were collected, of which 62% were fertilised via ICSI and 38% via IVF, with fertilisation rates equating to 64%. Eighty-one percent used partner sperm and 19% with donor sperm. Blastulation rates reached 64%. Out of all created blastocysts, 96% were biopsied for PGT-A.

### Clinical outcomes

A total of 805 euploid blastocysts were transferred in 795 FET cycles. A directive single blastocyst transfer policy is in place at this centre, however three patients underwent a double embryo transfer in their first euploid attempt, and seven in their second euploid transfer attempt. On average, patients underwent a mean of 1.3 ± 0.5 euploid blastocyst transfers within the study period. Of these, 99% were single euploid transfers.

Six-hundred and nine patients transferred 612 euploid blastocysts in their first euploid transfer attempt. Out of 609, 301 patients’ first euploid transfer ended in implantation failure (37%) or miscarriage (13%). One patient terminated their pregnancy (0.2%). One hundred and forty-nine patients underwent a second euploid blastocyst transfer with standard substituted cycle, transferring a total of 156 blastocysts. Fifty patients were referred for endometrial immune profiling after at least one failed euploid transfer. Patients referred for endometrial immune profiling (n=50) underwent between 1-4 failed euploid transfers and between a combined total of 1-9 failed untested and euploid transfers at this centre prior to referral. Of the final cohort that completed endometrial immune profiling (n=37), 43% of patients were diagnosed with an overactive profile, 27% a balanced profile, 16% an underactive profile and 14% a mixed profile. A personalised protocol was prescribed for FET according to the profile, as outlined in the methodology (**Table 2**). All patients who completed endometrial immune profiling and subsequent personalised FET protocol underwent single euploid blastocyst transfer.

**Table 2:** Endometrial immune profiling and subsequent treatment for patients referred between 2019-2024.

	%	Medication
Overactive	43% (16/37)	• Oestradiol tablets 8-10mg daily • Prednisolone 20mg daily • Progesterone pessaries 400mg 3x daily + intramuscular progesterone
Balanced	27% (10/37)	• Oral oestradiol 8-10mg daily • Progesterone pessaries 400mg 3x daily
Underactive	16% (6/37)	• Endometrial scratching • Oestradiol tablets 8-10mg daily • Progesterone pessaries 400mg 3x daily • Low-dose hCG during luteal support
Mixed	14% (5/37)	• Oestradiol tablets 8-10mg daily • Prednisolone 10mg daily • Progesterone pessaries 400mg 3x daily + intramuscular progesterone

**Figure 1:**
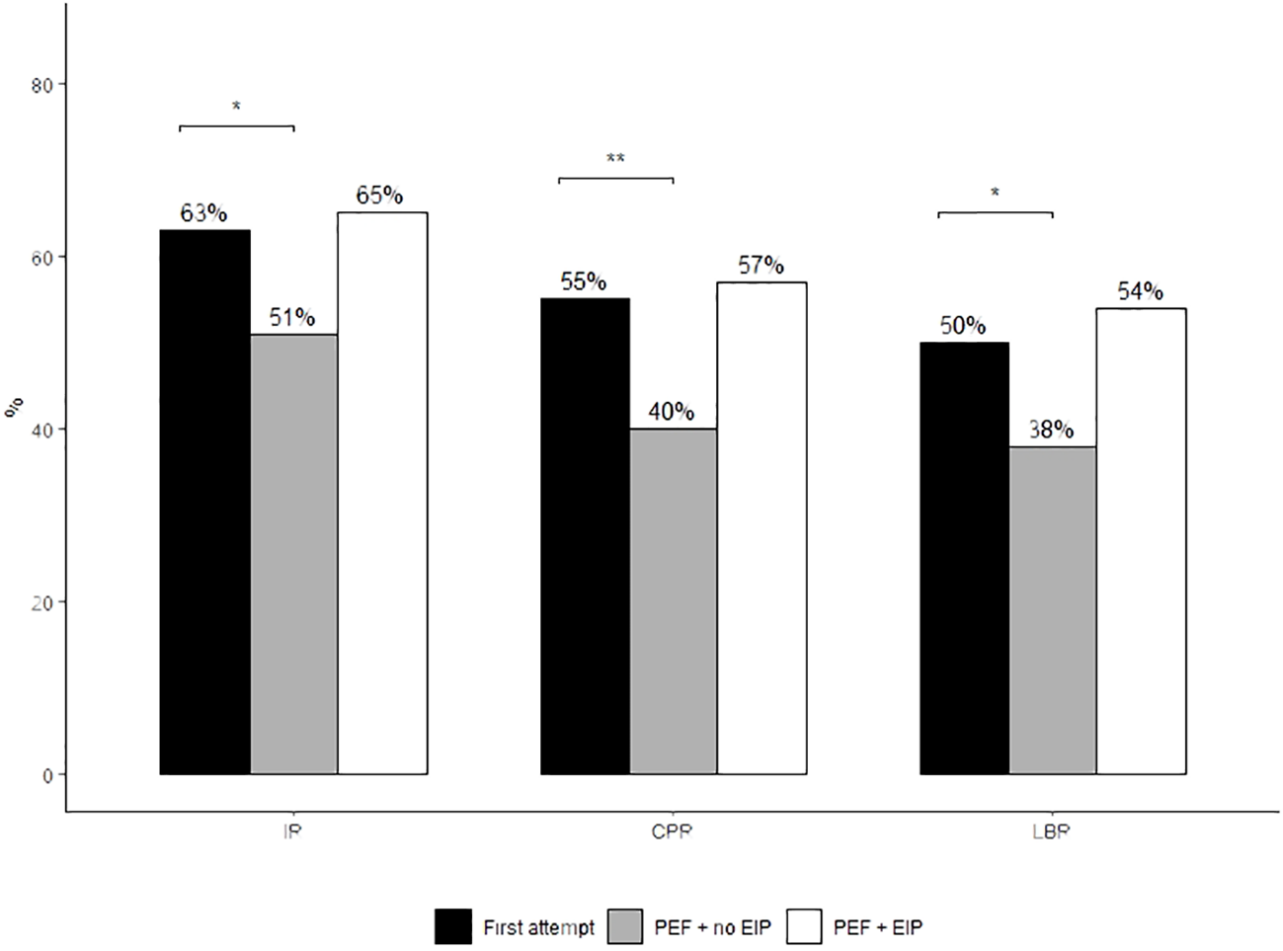
Implantation, clinical pregnancy and live birth rates of euploid FETs between study treatment groups. Proportions were compared using Fisher’s Exact test. *Post-hoc* comparisons were conducted using Tukey’s *post-hoc* test. *P<0.05 denotes statistical significance. **P<0.01. Only results with statistical significance are displayed on the graph. Absence of significance bars denotes no significant difference between groups.

Across all clinical outcomes, the PEF + EIP group achieved the highest rates (**Table 3**). This group achieved an implantation rate of 65%, a CPR of 57% with a final LBR of 54%. These rates were comparable with the first attempt group (65% vs 63%, *P*=1, 57% vs 55%, *P*= 0.9, 54% vs 50%, *P*= 0.7, respectively). This was the case despite the first attempt group having a significantly younger average oocyte age, greater number of embryos transferred per transfer and a greater proportion of embryos transferred morphologically graded as “BB” and above than both the PEF +EIP and no EIP groups (**Table 3**). In contrast, the PEF + no EIP group had a 14% lower implantation rate (51%) and a 16% lower LBR (38%) than the EIP group (**Figure 1**), despite having the youngest oocyte age across all groups and the greatest number of double embryo transfers, as well as no apparent differences in the proportion of “good quality” embryos transferred than the EIP group (**Table 3**). The IRs, CPRs and LBRs between the first attempt and PEF + no EIP groups significantly differed (63% vs 51%, *P=*0.02, 55% vs 40%, *P*=0.003, 50% vs 38%, *P*=0.02, respectively). Most likely due to small cohort size providing limited statistical power, no significant differences were found between the PEF + EIP group and the PEF + no EIP group, despite the largest differences in rates seen between these groups (IR; 65% vs 51%, *P*=0.2, LBR; 54% vs 38%, *P*=0.1, respectively). Miscarriage rates were comparable between groups, with a 2% lower miscarriage rate seen in the PEF + EIP group (PEF + EIP 11%; PEF + no EIP 13%; First attempt 13%).

**Table 3:** Clinical outcomes of euploid FETs between 2019-2024, per embryo transferred.

Clinical outcomes (per embryo transferred)	First euploid attempt (n=612)	Previous euploid failure + no immune profiling (n=156)	Previous euploid failure/s + immune profiling (n=37)	P-value*
Previous euploid failures [mean ± SD]	0 ± 0	1 ± 0	1.5 ± 0.8	*P*<0.001
Embryos transferred [mean ± SD]	1.0 ± 0.07	1.04 ± 0.2	1.0 ± 0	*P*<0.001
Embryos transferred graded BB and above, % (n)	81% (498/612)	73% (114/156)	73% (27/37)	*P*=0.04
Implantation Rate, % (n)	63% (387/612)	51% (80/156)	65% (24/37)	*P*=0.02
Clinical Pregnancy Rate, % (n)	55% (336/612)	40% (62/156)	57% (21/37)	*P*=0.002
Live birth Rate, % (n)	50% (306/612)	38% (59/156)	54% (20/37)	*P*=0.02

*P-value displayed is the unadjusted P-value across all three groups from a Kruskal Wallis test. Further pairwise *post-hoc* comparisons were also performed.

When exploring immune profiling results within each clinical outcome (live birth, implantation failure or miscarriage), there were no strong evident correlations noted between said two variables (**Table 4**).

**Table 4:** Immune profiling results by clinical outcome.

IP group (n=37)	Live birth (n=20)	Implantation failure (n=13)	Miscarriage (n=4)
Overactive, % (n)	45% (9/20)	46% (6/13)	25% (1/4)
Balanced, % (n)	30% (6/20)	14% (2/13)	50% (2/4)
Underactive, % (n)	20% (4/20)	8% (1/13)	25% (1/4)
Mixed, % (n)	5% (1/20)	31% (4/13)	0% (0/4)

Further, stratifying immune profiling results by patients with at least one previous euploid miscarriage (n=11) versus patients who only experienced previous euploid implantation failure/s (n=26) found a trend in the latter group of a slightly greater number of “overactive” profiles (36% vs 46%, respectively), and a greater number of “balanced” profiles in the previous miscarriage group (36% vs 23%, respectively). In terms of clinical outcomes, the previous miscarriage group achieved a 73% LBR following immune profiling and subsequent personalised protocol, whereas the IF group achieved a 46% LBR. Due to small sample sizes, statistics could not be performed to test for significance of these comparisons.

There were slightly less patients with a balanced profile who experienced implantation failure after immune profiling. Conversely, there were slightly more mixed profiles present in patients who experienced implantation failure after immune profiling. However, due to small numbers, statistical analysis could not be completed to draw robust conclusions from this data (**Table 4**).

## Discussion

In the present study, patients who underwent EIP after PEF achieved higher embryo implantation and LBRs than patients with PEF + no EIP. In comparison to the first euploid transfer attempt, the second conventional euploid transfer yielded significantly lower clinical outcomes. Outcomes between first euploid transfer attempts and the PEF + EIP group were comparable. Overall, this suggests EIP and personalised treatment plan may “restore” clinical outcomes back to rates seen in the first euploid attempt.

Transferring euploid-diagnosed embryos helps patients of advanced maternal age to conceive from the first attempt in IVF by eliminating aneuploidies (29–31). After the first euploid attempt, rates in the subsequent attempts decrease, for reasons that are unknown (32). One potential cause for failure is dysregulation in the endometrium, an observed symptom in those suffering from RIF or RM, particularly with aberrances in the recruitment, activation and regulation of key immunomodulatory markers that maintain the balance between embryo invasion and the mounted maternal immune response (10, 22). These aberrances are correlated with decreased implantation and LBRs (16, 17, 33). In this study, the PEF + EIP group achieved LBRs comparable to the first euploid transfer attempt. Contrastingly, the PEF + no EIP group had significantly lower clinical outcomes than the first attempt. This was the case despite the PEF + EIP group having a significantly older oocyte age, significantly more PEFs and only undergoing single embryo transfers.

Several studies have documented aberrant immune milieus in non-tested transfers, reporting correlations with aberrances in RIF (20, 34) and RM (19). However, when embryo ploidy is not controlled for, it can be difficult to discern whether failed embryo transfer is solely due to the endometrium. One study confirmed the presence of dysregulated cytotoxic immune cell pathways in women with failed euploid transfer (35). Another study analysed immune ratios and profiles in patients with previous euploid transfers, but with no subsequent treatment and transfers (36). Unlike these studies, the present study reports the usage of an endometrial immune diagnostic tool and subsequent treatment and with final treatment outcomes. In the present study, solely euploid embryo transfers were included to isolate the role of the endometrium in embryo implantation as best as possible. To the author’s knowledge, this is the first study investigating endometrial immune profiling with confirmation of embryo ploidy for previous failures and subsequent treatment.

Possibly reflecting a lack of statistical power, no significant differences were seen between clinical outcomes of the PEF + no EIP group and the PEF + EIP group, despite the largest differences in rates seen between these groups. While the present study’s intervention cohort is too small to draw robust conclusions, these findings show potential trends that future studies may endeavour to explore on a larger scale.

Recent studies and extrapolated data (37, 38) have developed a narrative that RIF is a very rare phenomenon that can essentially be avoided by serial transfer of euploid embryos. These datasets predict that after three euploid embryo transfers, 93% of patients can achieve a LB and after five euploid embryo transfers, a predicted cumulative LBR of 98%. Moreover, they report implantation rates being sustained through serial euploid transfers. The further implication of these studies is therefore that the endometrium is not a significant determining factor for successful euploid transfer.

However, this extrapolated data is calculated under optimistic assumptions, such as that no patient discontinues treatment and that patients have sufficient euploid embryos to complete five transfers. The real-life challenge is that very few patients receive 3-5 euploid-diagnosed embryos, as well as the financial, emotional and physical burdens that repeated cycles could inflict. Thus, in scenarios where an endometrial factor is suspected, the available solution is rarely eradication of the endometrial factor through multiple euploid transfers, but rather to correct any identifiable endometrial factor and achieve a LB within a confined attempt limit.

Indeed, in our clinical setting, only 22% of cycles from patients over 38 years old had at least one embryo diagnosed as euploid, averaging 0.9 euploid embryos per cycle. Cumulative live birth rates after three euploid transfers for this age group is 66%, and after five reaches only 68% [unpublished data]. This is comparable with a recent report comparing cumulative LBRs in IVF cycles with euploid embryos (36). To conclude, why 20-30% of euploids fail to implant, and why euploids in subsequent transfers achieve lower clinical outcomes, remains unanswered. And while this remains so, factors such as the endometrium cannot be entirely ruled out as a possible reason for failure when embryo factors appear controlled-for.

The primary limitation of this study is the small cohort size that meant there was lack of statistical power to detect a significant difference between the EIP group and other groups. The second limitation is its retrospective nature, which means there are known and unknown confounders present throughout the data, such as variation in patient & cycle variables and differences in sample n for each study arm. Although previous studies have confirmed immune profile correction following treatment (19, 24, 39, 40), there were no repeat biopsies performed after personalised treatment in this study.

However, the strengths of this study include the standardisation of all treatments through a single centre with consistent policies applying to PGT technique and the platform used throughout the period reported. It is also one of the first studies investigating endometrial immune profiling within euploid transfers to control for embryo factor with treatment. Thirdly, in context of the existing literature, this study provides data that challenges current narratives regarding the role of the endometrium in RIF, and the widespread practice of empirical interventions provided without any attempt at an ‘endometrial diagnosis’.

## Conclusions

Overall, these findings suggest that profiling of the endometrial immune milieu, with adjusted treatment cycles in those with dysregulated profiles, may improve chances of conception in subsequent euploid attempts for specific patient subsets, and may restore rates back to those seen in the first euploid transfer attempt. While only able to report initial trends, these findings hold promise that endometrial immune assessment could improve the likelihood of achieving a successful pregnancy for some patients who are struggling to conceive with a euploid embryo. Future work is needed to expand cohort size and reinforce the trends reported here with robust statistical conclusions.

## Data Availability

Data can be made available on reasonable request to the corresponding author. Requests to access these datasets should be directed to mona.rahmati@londonwomensclinic.com.
